# Numerical Demonstration of 800 Gbps WDM Silicon Photonic Transmitter with Sub-Decibel Surface-Normal Optical Interfaces

**DOI:** 10.3390/mi13020251

**Published:** 2022-02-02

**Authors:** Zanyun Zhang, Meixin Li, Kaixin Zhang, Tianjun Liu, Beiju Huang, Hao Jiang, Yilin Liu, Qixin Wang, Jiaming Xing, Bo Yuan, Hongwei Liu, Pingjuan Niu

**Affiliations:** 1 School of Electronic and Information Engineering, Tiangong University, Tianjin 300387, China; 2031070940@tiangong.edu.cn (M.L.); 1931075515@tiangong.edu.cn (K.Z.); 2031070960@tiangong.edu.cn (T.L.); 2130070816@tiangong.edu.cn (H.J.); 2131070986@tiangong.edu.cn (Y.L.); 2131070964@tiangong.edu.cn (Q.W.); 2131070990@tiangong.edu.cn (J.X.); 2131070982@tiangong.edu.cn (B.Y.); liuhongwei@tiangong.edu.cn (H.L.); niupingjuan@tiangong.edu.cn (P.N.); 2 Tianjin Key Laboratory of Optoelectronic Detection Technology and Systems, Tiangong University, Tianjin 300387, China; 3 State Key Laboratory on Integrated Optoelectronics, Institute of Semiconductors, Chinese Academy of Sciences, Beijing 100083, China; bjhuang@semi.ac.cn

**Keywords:** WDM silicon photonic transmitter, perfectly vertical grating coupler, interleaved AMMI

## Abstract

We propose and numerically demonstrate an 800 Gbps silicon photonic transmitter with sub-decibel surface-normal optical interfaces. The silicon photonic transmitter is composed of eight silicon Mach–Zehnder optical modulators and an interleaved AMMI WDM device. This WDM device comprises two 1 × 4 angled MMI and a Mach–Zehnder interferometer (MZI) optical interleaver with an apodized bidirectional grating which has about −0.5 dB coupling loss. Both the Mach–Zehnder electro-optical modulators and MZI optical interleaver regard the bidirectional grating coupler as vertical optical coupler and 3-dB power splitter/combiner. By importing the S-parameter matrices of all the components which have been carefully designed in simulation software, the circuit-level model of the optical transmitter can be built up. On this basis, the static and dynamic performance characterization were carried out numerically. For NRZ modulation, the optical transmitter exhibits the overall optical loss of 4.86–6.72 dB for eight wavelength channels. For PAM4 modulation, the optical loss is about 0.5 dB larger than that of NRZ modulation, which varies between 5.38–7.27 dB. From the eye diagram test results, the WDM silicon photonic transmitter can achieve single channel data transmission at 100 Gb/s NRZ data or 50 GBaud/s PAM4 symbol rate with acceptable bit error rate.

## 1. Introduction

The massive amount of data movement generated by application-driven traffic demand such as HD video streaming, cloud-based computing and storage, Internet-of-Things, and 5G applications is forecasted to reach 28.8 zettabytes by 2022, and it will grow at a compound average rate of 30% per year. To meet this demand, innovations of interconnect are urgently needed to provide denser connectivity with high bandwidth, low latency, and high energy efficiency in a cost-effective manner, especially in the datacenters and high-performance computing systems [1,2]. Optical interconnects are emerging as a viable solution over copper wires for board-to-board data communication [3]. With the ever-increasing demand for higher bandwidth, advanced optical interconnects are progressively penetrating to shorter distances such as inter-chip and intra-chip applications for processors and switches [4].

Silicon photonics is considered an economical candidate for optical interconnects by taking advantage of the existing mature CMOS technology to offer unprecedented bandwidth scalability using spatial division multiplexing [5], wavelength-division multiplexing [6], polarization-division multiplexing [7], and advanced modulation formats. Normally, spatial division multiplexing is quite suitable for the short reach optical interconnect applications, i.e., 500 m, while wavelength-division multiplexing is commonly used for targeted range of a few kilometers. Especially, the coarse wavelength-division multiplexing (CWDM) is utilized in today’s silicon photonic products such as the 100G CWDM4 QSFP28 optical transceiver and the recently released 400 G optical transceiver using 4-level pulse amplitude modulation (PAM4) by Intel [8]. Recently, there are also many other demonstrations of silicon optical transceivers which are further extending the data transmission speed to more than 400 Gb/s [9,10,11]. Towards 800 G silicon photonic optical modules, there are many viable solutions such as PSM 4 × 200 G, PSM 8 × 100 G, and WDM 8 × 100 G [12,13,14]. There are also a few demonstrations of Terabit/s optical I/O chiplets integrated with multiple wavelength lasers and ASICs [15,16], which show the great potential for large capacity optical communication using the WDM techniques.

As an important building block of the silicon photonic transceivers, silicon optical transmitters achieved great success during the past few years [17,18,19]. Currently, silicon optical transmitters are more densely integrated and most of the performance metrics, i.e., data capacity and power efficiency, are comparable to its III–V alternatives [20]. However, the rapid commercialization of silicon photonic products is still hindered by two main drawbacks. One is the relatively large optical loss of the silicon photonic circuits, which is mainly contributed by the fiber-chip coupling loss and the on-chip insertion loss of the photonic devices. As the on-chip optical amplification function is not currently available, the power budget of the silicon photonic circuits is of paramount importance and directly related to the quality of optical communications. Thus, high efficiency grating couplers and low-loss photonic devices are urgently needed. Another main roadblock would be the viable and cost-effective on-chip laser source for silicon photonic circuits. Although heterogeneous integrated III–V lasers on silicon platform have been successfully demonstrated, but issues around the CMOS process compatibility, yield, and reliability remain to be resolved [21,22,23]. In recent years, hybrid integration of VCSELs is emerging as a promising solution as a hybrid laser source via grating coupling. Unfortunately, the tilting coupling scheme of conventional grating couplers leads to a large obstacle for the coplanar integration of VCSEL and silicon photonic chips. Although many attempts are proposed to solve this problem, such as using a high-contrast grating as the bottom reflector [24], adding a SU8 prism above the grating coupler [25], and controlling the reflow of the solder ball deposition [26], these techniques suffer from low coupling efficiency, complex fabrication process and high cost. To decrease packaging complexity, high-efficiency, perfectly vertical grating couplers would be the key to promoting the integrated hybrid silicon-based VCSELs. Besides, perfectly vertical grating coupling [27,28,29,30], which enables fiber packaging without angle polishing, can be very useful to decrease the fiber packaging cost [31,32,33].

In this paper, an 800 Gbps WDM silicon photonic transmitter with surface-normal optical interfaces is proposed and numerically demonstrated. This optical transmitter is comprised of eight silicon Mach–Zehnder optical modulators and an 8-channel interleaved angled MMI (AMMI) (de)multiplexer, all of which contain a high-efficiency bidirectional grating coupler functioning as both the perfectly vertical grating coupler and 3-dB power splitter. To improve the coupling performance, an apodized overlay grating structure with subwavelength gratings is proposed and carefully designed. Based on the simulation and modeling work of the grating coupler, the optical modulator and the interleaved AMMI, the system performance was analyzed with the circuit simulation model using Ansys Lumerical interconnect. The data transmission experiment was carried out and the bit error test results were analyzed as well.

## 2. System Design Overview

**Figure 1:**
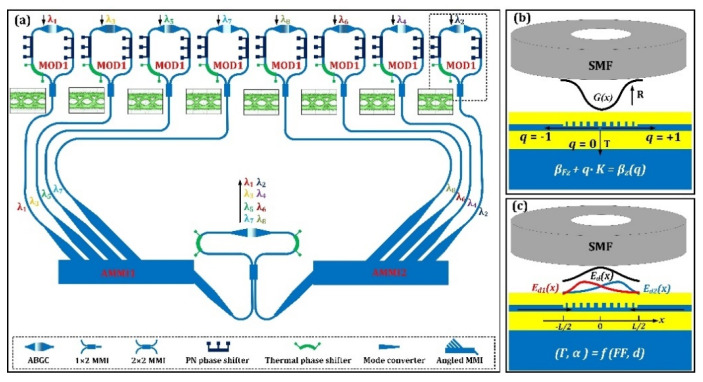
(**a**) Schematic of the proposed WDM silicon photonic transmitter. (**b**) Schematic of the grating coupler interfacing with an external input single mode fiber. (**c**) Schematic of the grating coupler interfacing with an external output single mode fiber.

Figure 1a shows the schematic of the proposed WDM silicon photonic transmitter with surface-normal optical interfaces for optical input and output. Eight silicon Mach–Zehnder optical modulators based on bidirectional grating couplers are utilized to convert the electrical data to the optical domain with eight different wavelengths. For wavelength division multiplexing, an interleaved AMMI is utilized which is comprised of two 1 × 4 AMMIs and a bidirectional grating based MZI optical interleaver. As a key component, the bidirectional grating couplers are designed for perfectly vertical coupling and the structure is carefully optimized by the techniques of grating apodization and subwavelength grating engineering. Thus, the eight optical signals with different wavelengths are launched in-plane and coupled out-of-plane by the perfectly vertical grating couplers. The surface-normal optical interfaces make this silicon photonic transmitter chip very promising for low-cost fiber packaging.

## 3. Component Design and Modeling

### 3.1. Apodized Bidirectional Grating Coupler

For the proposed silicon photonic transmitter, the apodized bidirectional grating coupler design is very important as it largely affects the WDM optical transmitter performance. Ideally, the perfectly vertical grating coupler should feature high efficiency, low parasitic reflection, large optical bandwidth and ease of fabrication. However, it is hard to fulfill all the requirements simultaneously. Here we present a grating design which fits well with the design requirement. The structural view and working principle of the grating coupler is depicted in Figure 1b,c. According to the grating coupling theory [34], the grating structure design should fit the Bragg condition for perfectly vertical coupling:

(1) $$\beta_{Fz}+q\cdot K=\beta_{z}\left(q\right)$$

where *β_Fz_* represents the *z*-axis (the waveguide transmission direction) component of the propagation constant of the single-mode fiber fundamental mode, q indicates the diffraction order, *K* is the absolute value of the grating vector *K* which equals to *2π/Λ*, *β_z_*(*q*) represents the *z*-axis component of the propagation constant of the *q^th^* diffraction order mode. For bidirectional grating coupling, *β_Fz_* equals to zero. Designed as a second-order grating, the bidirectional GC supports diffraction orders from −1 to 1; diffraction order modes of −1 and 1 correspond to the fundamental waveguide modes of two opposite directions; diffraction order modes of 0 corresponds to the substrate leakage power (*T*) and the upward reflection power (*R*). Thus, the fiber-to-chip coupling efficiency can be expressed as:

(2) $$CE=\left(1-R-T\right)\cdot \gamma \left(TE_{0}\right)$$

where *γ*(*TE*_0_) represents the fundamental *TE* mode coupling ratio (the optical power coupled into the *TE* waveguide fundamental mode divided by the total optical power of waveguide coupling). According to our simulations, the bidirectional grating couplers with waveguide thickness of 220 nm corresponds to *γ*(*TE*_0_) of about 0.99, which means almost all the waveguide coupling are in *TE* fundamental mode. From the view of out-of-plane coupling (as depicted in Figure 1c, the working principle can be expressed as follows. Firstly, two grating diffraction modes can be excited by the two incident waveguide modes in opposite directions, and the two diffracted electric fields (*E*_*d*1_(*x*) and *E*_*d*2_(*x*)) can be given by the following equations:

(3) $$\{\begin{array}{c} E_{d1}\left(x\right)\propto \sqrt{2\alpha \Gamma }e^{-\alpha \left(x+\frac{L}{2}\right)}\\ E_{d2}\left(x\right)\propto \sqrt{2\alpha \Gamma }e^{\alpha \left(x-\frac{L}{2}\right)} \end{array}$$

where *α* is the coupling strength of the grating coupler, Γ stands for the directionality of the grating coupler, *L* is the lateral length of the grating. If we consider the two diffraction modes to be in phase, the final grating diffraction mode (*E_d_(x)*) can be obtained from the constructive interference of the two excited diffraction fields:

(4) $$E_{d}\left(x\right)=\sqrt{\frac{E_{d1}^{2}\left(x\right)+E_{d2}^{2}\left(x\right)}{2}}$$

From Equations (3) and (4), it can be deduced that:

(5) $$E_{d}\left(x\right)=\sqrt{\alpha \Gamma e^{-\alpha L}\left(e^{-2\alpha x}+e^{2\alpha x}\right)}$$

The fiber mode field *G*(*x*) can be expressed as the following equation:

(6) $$G\left(x\right)\propto \frac{1}{\sqrt{w_{0}}}exp\left(-\frac{{\left(x-\mu \right)}^{2}}{w_{0}^{2}}\right)$$

As a one-dimensional approximation, the coupling efficiency can be calculated through the overlap integral of the diffraction mode and the single-mode fiber mode:

(7) $$CE={\left|\int_{\mu -\frac{D}{2}}^{\mu +\frac{D}{2}}E_{d}\left(x\right)G\left(x\right)dx\right|}^{2}$$

**Figure 2:**
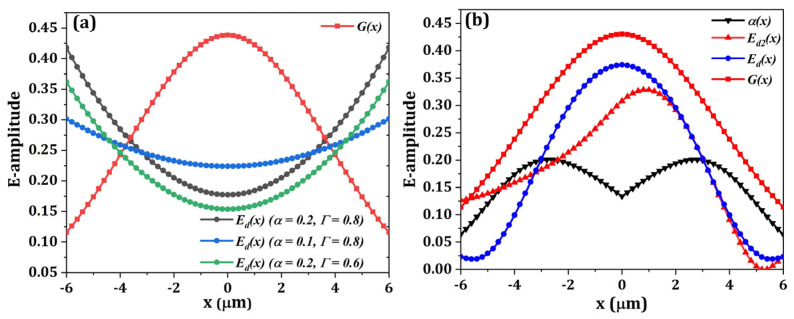
The comparison between the Gaussian field and the diffraction field of (**a**) uniform bidirectional grating couplers with different coupling strength and grating directionality. (**b**) an apodized bidirectional grating coupler with engineered coupling strength distribution.

From Equation (5), it is clear that the diffraction field pattern of a bidirectional GC is mainly related to the coupling strength distribution of the grating structure while the amplitude of the E-field is jointly influenced by the grating directionality and the coupling strength distribution. This relationship can be seen from the comparison between the calculated Gaussian field and the bidirectional grating diffraction field shown in Figure 2a.

Clearly, there is a big mode mismatch between the grating diffraction mode and fiber mode for a uniform bidirectional grating design. Although a weak grating design (with small α) can help improve the matching efficiency, it is still impossible to obtain a Gaussian-like diffraction field with uniform grating design. As the coupling strength α and the grating directionality Γ are both functions of the grating filling factor (FF) and etching depth (d), the grating radiation mode can be reshaped by engineering the grating filling factors and etching depth of each grating cells. To improve the mode matching, apodized grating design is utilized for tailoring the grating coupling strength distribution. As shown in Figure 2b, a carefully designed coupling strength distribution *α*(*x*) can result in a Gaussian-like diffraction field without breaking the grating symmetry. By carefully designing the grating pitches and filling factors, the mode matching can be improved, and the parasitic reflections can be suppressed.

**Figure 3:**
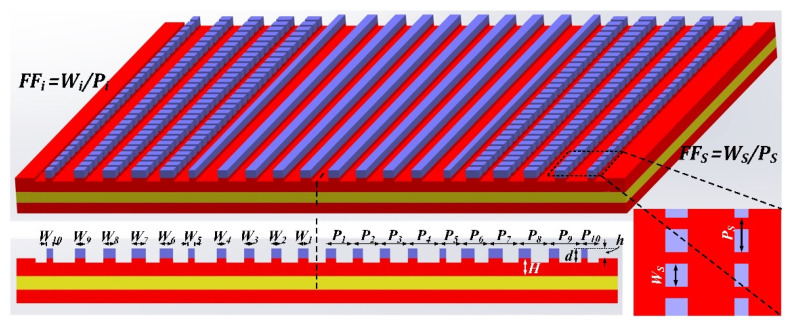
The schematic diagram of bidirectional grating coupler design.

Based on the working principle above, we proposed and designed a highly efficient bidirectional grating coupler which combines the technology of grating apodization, overlay technique and subwavelength gratings. Figure 3 shows the structural view of the proposed grating coupler. As can be seen, the grating coupler is designed as central symmetric with 20 grating pitches, of which the inner 10 pitches are standard grating cells while the outer 10 pitches are subwavelength engineered ones. For performance optimization of the optical transmitter, two grating designs were utilized, and the specific design parameters are shown in Table 1.

Due to the bandwidth limitation of the grating couplers, two GCs (ABG_1_ and ABG_2_) are designed with different central coupling wavelengths, for decreasing the internal Fabry–Perot reflection [35] and improving the channel uniformity. As can be seen, the GCs are designed with waveguide height (H) of 220 nm, polysilicon overlayer height (h) of 160 nm covering the grating region and etching depth (d) of 230 nm in total. The grating structure parameters (grating pitches (P_i_), grating filling factors (FF_i_) and filling factor of subwavelength gratings (FF_s_)) are carefully engineered for the optimal device performance with a series of numerical calculations by the Finite-difference-time-domain (FDTD) simulation method.

**Figure 4:**
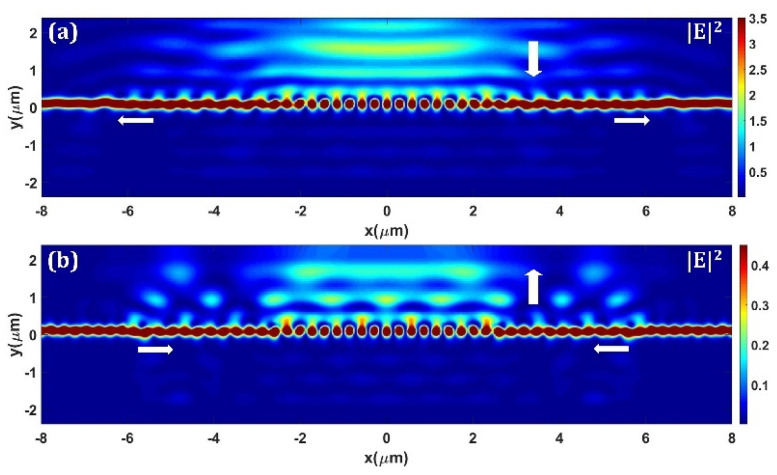
Calculated optical field intensity profile of (**a**) in-plane coupling and (**b**) out of plane coupling with the ABG_1_.

The design procedure of the GCs is as follows. First, the grating directionality and coupling strength are calculated as a function of grating filling factor, respectively. According to the mode matching theory of grating coupling, the grating coupling strength profile for a quasi-Gaussian diffraction field can be calculated. Second, the grating parameters are initially settled based on the calculation results. In addition, the subwavelength gratings are introduced to replace the grating segments with small filling factors, according to the effective medium theory (EMT) [36]. Finally, the grating couplers are optimized using particle swarm algorithm with 2D FDTD method and then verified using 3D FDTD method. Figure 4a,b show the calculated optical field intensity profile of the bidirectional GC (ABG_1_) with the fiber incidence coupling and the waveguide incidence coupling, respectively. As can be seen, the coupler is highly efficient, and the parasitic reflections and substrate leakage power are greatly suppressed.

**Figure 5:**
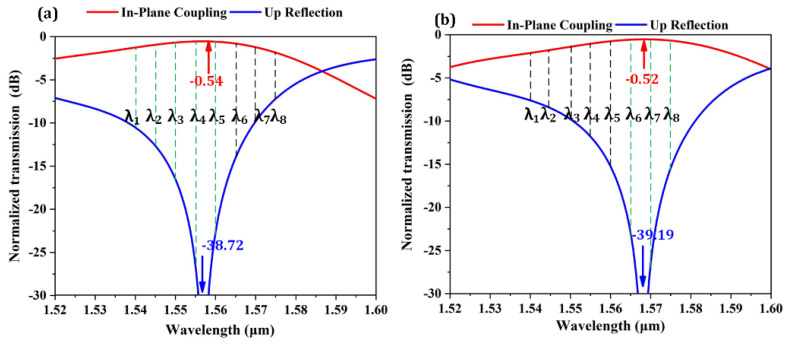
(**a**) Coupling efficiency and reflection spectra of ABG_1_. (**b**) Coupling efficiency and reflection spectra of ABG_2_.

Figure 5a shows the coupling efficiency and upward reflection spectra of ABG_1_. The central coupling wavelength is designed to be 1557 nm and the peak coupling efficiency reaches 88.28% (−0.54 dB). The 1-dB bandwidth of ABG1 is about 37 nm. To obtain a channel nonuniformity below 1dB, a WDM channel spacing of 5 nm is utilized for our transmitter design. As depicted in Figure 5a, eight wavelength channels are distributed in the central wavelength range with high coupling efficiency and low upward reflection. For the optical transmitter, the upward reflection of the GCs should be suppressed to avoid strong F-P resonance. To compensate for the bandwidth limitation of the grating coupler, another grating coupler (ABG_2_) with a central wavelength of 1570 nm is designed for the optical modulators of the last three wavelength channels (*λ*_6_–*λ*_8_). Figure 5b shows the simulated coupling performance of ABG_2_. The maximum coupling efficiency is 88.54% (−0.52 dB), the 1-dB bandwidth is 38 nm. As can be seen, all the wavelength channels are located in the low-reflection wavelength region. Table 2 shows the comparison between our coupler design and other state-of-the art fiber-chip grating couplers [37,38,39,40,41,42,43], from which it can be seen that our coupler exhibits the advantages of high coupling efficiency, ease of fabrication and surface-normal optical coupling. 

### 3.2. Mach–Zehnder Optical Modulators

**Figure 6:**
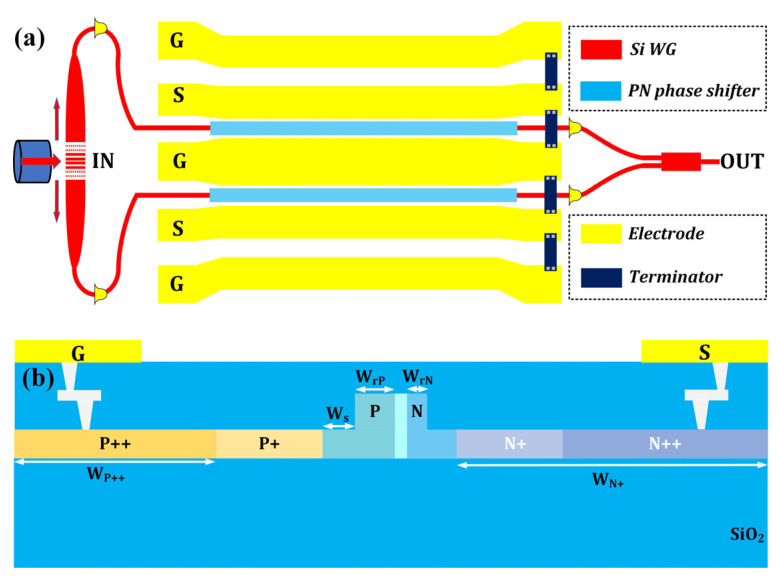
(**a**) The schematic diagram of the silicon Mach–Zehnder optical modulator based on bidirectional grating coupler. (**b**) The schematic cross-sectional view of the P-N phase shifter.

To construct a Mach–Zehnder optical modulator [44], the subwavelength apodized bidirectional grating coupler is used as a power splitter at the input port, while a 1 × 2 MMI coupler is utilized for optical power combining at the output port. A length difference Δ*L* is introduced to form an asymmetric MZI. The schematic diagram of the optical modulator is shown in Figure 6a, and the cross-sectional view of the P-N phase shifter is shown in Figure 6b. As can be seen, a dual-drive push-pull operation scheme is utilized in the modulator design and on-chip terminator can be integrated for impedance matching. The cross-sectional view of the P-N phase shifter design is depicted in Figure 6b. The phase shifter is based on a ridge waveguide structure with width of 500 nm, height of 220 nm and slab thickness of 70 nm. Six different doping conditions are adopted to form the phase shifter. To improve the modulation efficiency, the P-N junction located in the waveguide rib is fully optimized with Electro-Optical simulations. As a result, the P-type doped region in the rib is 300 nm in width and the N-type doping region is 100 nm in width. This means an intrinsic layer of 100 nm exists between P and N doping regions. This helps decrease the capacitance and improve the modulation speed. The P+ and N+ regions are medium-doped regions, which are located 250 nm away from the edge of the ridge waveguide to achieve a balanced performance between optical loss and modulation speed. P++ and N++ dopings are used for ohmic contact with the metal electrode.

**Figure 7:**
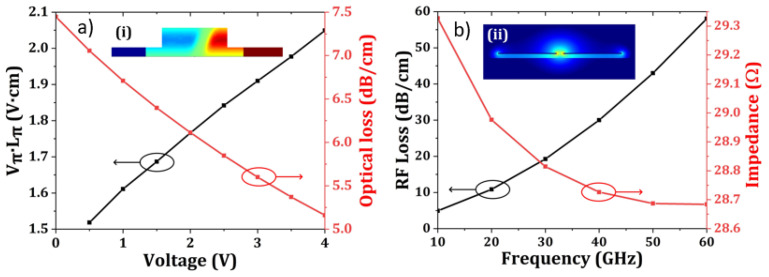
The simulation results of PN phase shifter. (**a**) Modulation efficiency (dot and black line) and optical waveguide transmission loss (dot and red line) with different applied voltages. The inset picture shows the simulated doping profile of the PN phase shifter. (**b**) Microwave loss (dot and black line) and impedance of the travelling wave electrode (dot and red line). The inset shows the microwave mode profile.

Table 3 listed all the design parameters of the PN phase shifter, which are used in the simulations of the optical modulator and the whole optical transmitter. To evaluate the performance of the phase shifter, we calculate the carrier density distribution change and PN junction capacitance change under different voltages using the commercially available software of Ansys Lumerical Charge. Then by importing the data to the optical waveguide and coplanar waveguide electrode simulations, the modulation efficiency, the optical loss and the characteristics of the travelling wave electrode can be obtained, which are depicted in Figure 7. The modulation efficiency and the optical loss are about 1.6 V·cm and 6.7 dB/cm with a bias voltage of 1 V. The microwave transmission loss and impedance of the travelling wave electrode (TWE) is shown in Figure 7b. The modulator speed is mainly decided by the 3-dB E-O bandwidth, which can be calculated by the following equation:

(8) $$EO_{S_{21}}=10log\frac{{\left|S_{21}\right|}^{2}-2\cdot \left|S_{21}\right|\cdot \cos\left(\beta_{opt}^{u}\cdot l\right)+1}{{\left(\ln\left|S_{21}\right|\right)}^{2}+{\left(\beta_{opt}^{u}\cdot l\right)}^{2}}$$

(9) $$\beta_{opt}^{u}=\frac{\omega_{m}}{c}\left(n_{\mu }-n_{opt}\right)$$

In the formula, *S*_21_ represents the transmission coefficient of the traveling wave electrode scattering matrix, $\beta_{opt}^{u}$ represents the velocity matching between the microwave and light wave, *L* is the length of the traveling wave electrode, *ω*_m_ is the angular frequency of the modulator, *c* is the speed of light, *n_u_* is the microwave refractive index, *n_opt_* is the refractive index of light. It can be known from the formula that the electro-optical bandwidth is related to $\beta_{opt}^{u}$, *S*_21_ and the length of the modulator. The refractive index of light wave *n_opt_* is affected by waveguide shapes, doping profiles of the phase shifters. The refractive index of the microwave *n_u_* is mainly related to the electrode geometry and capacitance of the PN junctions. Clearly, there are many factors affecting the 3 dB E-O bandwidth and performance tradeoffs are always faced when designing the modulators. For balanced performance between insertion loss, modulation speed and modulation depth, the phase shifter length is chosen as 2 mm. According to our simulation results, the corresponding 3-dB E-O bandwidth of the optical modulator is 35.83 GHz. To achieve impedance matching, on-chip TiN terminator of 30 Ω is utilized in our simulations. 

**Figure 8:**
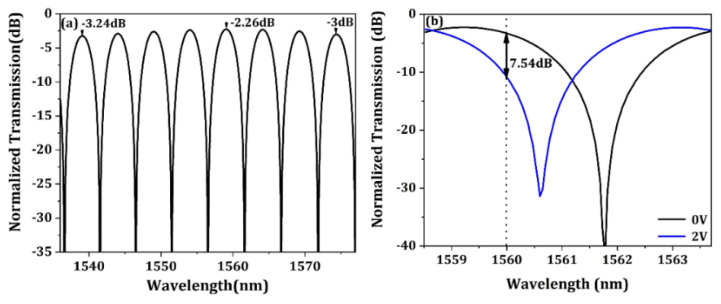
(**a**) The normalized transmission spectrum of MZI. (**b**) The optical transmission spectrum varies with voltage.

The transmission spectra of the silicon Mach–Zehnder interferometer can be expressed as follows:

(10) $$T\left(\lambda \right)=\frac{C\left(\lambda \right)}{2}\cdot \left[1+2\sqrt{S\left(\lambda \right)(1-S\left(\lambda \right)}\cdot \cos\left(\varphi \left(\lambda \right)\right)\right]$$

(11) $$\varphi \left(\lambda \right)=\frac{2\pi N_{effw}}{\lambda }\cdot \Delta L+\frac{2\pi N_{effg}}{\lambda }\cdot 2\Delta x$$

where *C*(*λ*) represents the total coupling efficiency of the bidirectional grating coupler at a specific wavelength, *φ*(*λ*) represents the optical phase difference between the two optical arms of the MZI, *N_effw_* and *N_effg_* represent the effective refractive index of single mode waveguide and the grating structure, respectively. *S*(*λ*) represents the grating splitting ratio, which is defined as the optical power splitting into one waveguide arms divided by the total power coupling of the two waveguide arms. Specially, *S*(*λ*) equals to 0.5 when the fiber is perfectly aligned in the grating center. Δ*L* and Δ*x* represent the waveguide arm length difference and the lateral fiber misalignment with respect to the grating center, respectively. However, in order to obtain the optical transmission of the optical modulator, the waveguide loss and optical loss caused by doping cannot be ignored. Therefore, we use a circuit model to calculate the transmission spectra of the optical modulator. In the optical domain, the scattering parameter matrix of the bidirectional grating coupler and the MMI coupler are extracted from the FDTD simulations. Based on the compact model of those optical elements, the circuit model of the Mach–Zehnder interferometer is built up. In the electrical domain, the transmission line model of the travelling wave electrode is formed by incorporating the P-N junction parameters such as the series resistance and capacitance obtained from the commercially available software charge. Thus, both the static and dynamic response of the optical modulator to electrical signals can be calculated. Figure 8a,b shows the normalized transmission spectrum of the MZ optical modulator and the spectra response with driving voltage of 2 V, respectively. As can be seen, the minimum insertion loss is only 2.26 dB, which includes the bidirectional grating coupling loss of 0.52 dB, the MMI coupler loss of 0.15 dB, the phase shifter optical loss of 1.5 dB and the rest of waveguide transmission and bending loss. From Figure 8b, it can be seen that the static extinction ratio (ER) of 7.54 dB can be obtained with a working wavelength of 1560 nm which is perfectly matched to one of the WDM channels. The excess optical loss for the operation wavelength is about 1.2 dB. 

In order to match the modulator working wavelengths with the WDM channels, the free spectra range (*FSR*) of the MZI is designed as 5 nm. According to the optical theory, the FSR is related to the arm length difference of the MZI:

(12) $$FSR=\frac{\lambda_{1}\cdot \lambda_{2}}{N_{gw}\cdot \Delta L}\approx \frac{\lambda_{0}^{2}}{N_{gw}\cdot \Delta L}$$

where *λ*_0_ is the vacuum wavelength of 1550 nm, and *N_gw_* is the group refractive index of the single-mode ridge waveguide, which is set to 3.9629 in the simulation, and Δ*L* is calculated to be approximately 121.12 μm by the above formula.

**Figure 9:**
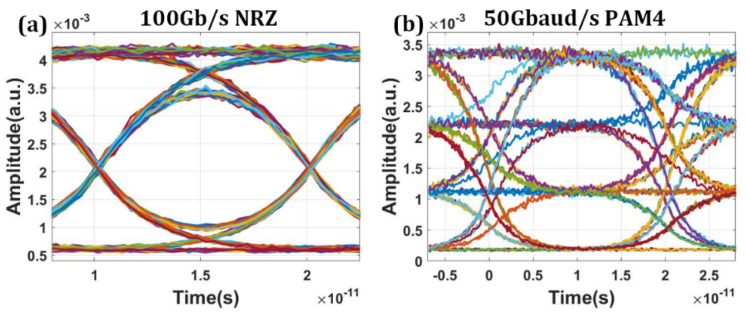
Simulated eye diagram results of eight channels of silicon-based electro-optical modulator at 100 Gbit/s. (**a**) The eye diagram obtained by the modulator during 100 Gbit/s NRZ signal transmission. (**b**) The eye diagram obtained by the modulator during 50 Gbaud/s PAM4 signal transmission.

The dynamic performances of the MZ modulators were investigated with the data transmission simulation setup, which included the compact model of the modulators, the continuous wave laser, the PRBS generator, the NRZ/PAM4 pulse generator and eye diagram analyzer. Figure 9a,b show the eye diagrams at 100 Gb/s of OOK modulation and 50 Gbaud/s of PAM4 modulation. The peak-to-peak driving voltage of OOK and PAM4 modulation are set at 2 V. To improve the signal integrity and linearity, the bias condition of the working wavelength and the four voltage levels of PAM4 modulation are carefully chosen. The clear open eyes imply that single lane of 100 Gb/s can be supported with both OOK and PAM4 modulation formats. The corresponding ER are about 6.8 dB and 4.9 dB, respectively. 

### 3.3. Interleaved AMMI Multiplexer/Demultiplexer

The wavelength division multiplexing function is carried out by an interleaved AMMI device [45,46,47], which is schematically shown in Figure 1a. Notably, the device contains a bidirectional grating coupler which functions as the perfectly vertical grating coupler as well as the power splitter/combiner. From the view of optical filtering, this device can be seen as a three-stage optical filter. At the first stage, the grating coupler provides a broadband wavelength window that allows all the wavelength signals to be coupled into the waveguide plane. As the second stage, the MZI functions as a comb filter with two complementary outputs that divides the optical signals into two groups with odd (*λ*_1_, *λ*_3_, *λ*_5_, *λ*_7_) and even channel numbers (*λ*_2_, *λ*_4_, *λ*_6_, *λ*_8_), respectively. Lastly, two 1 × 4 AMMIs cascaded with the MZI demultiplex the two signal groups into separate wavelength channels, respectively. 

The key of designing such a *WDM* device is to achieve a perfect matching between the MZI filter and the AMMI filters, which means firstly the free spectral range (*FSR*) of the MZI should be equal to the channel spacing of the two AMMIs:

(13) $$\Delta \lambda_{\mathrm{MZI}}=\Delta \lambda_{\mathrm{AMMI}}$$

and the design rules of the AMMIs can be expressed as the following equations:

(14) $$L_{i}=\frac{4N_{eff}W_{m}^{2}}{\lambda_{i}}$$

(15) $$\Delta \lambda_{min}>\frac{W_{a}+x_{min}}{4N_{eff}sin\theta }{\left(\frac{\lambda_{i}}{W_{m}}\right)}^{2}$$

(16) $$N_{max}<\frac{2W_{m}cos\theta }{W_{a}}$$

**Figure 10:**
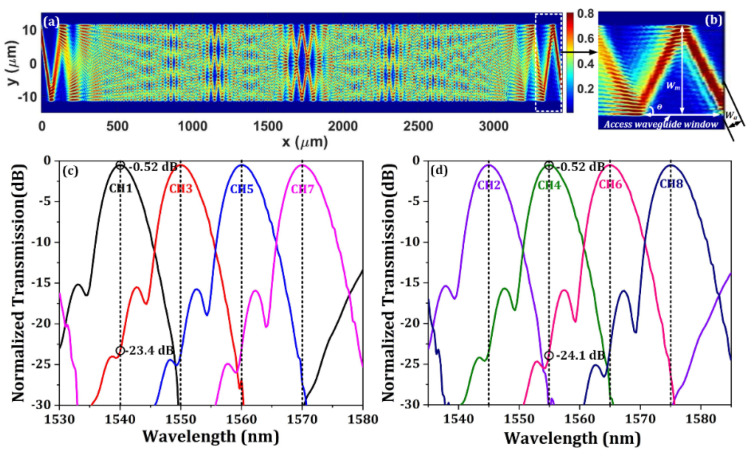
(**a**) The calculated electric field intensity distribution of AMMI at the wavelength of 1550 nm. (**b**) The zoomed-in field profile showing the access waveguide window on the sidewall of the multimode waveguide. (**c**) The calculated transmission spectra of the 1 × 4 AMMI1. (**d**) The calculated transmission spectra of the 1 × 4 AMMI2.

Equation (14) represents the inverted self-imaging axial positions (*L_i_*) for different wavelength channels (*λ_i_*) of the AMMIs, where *N_eff_* represents the effective index of the multimode waveguide fundamental mode, *W_m_* is the width of the multimode interference waveguide. Equations (15) and (16) represent the design limitations of the AMMIs on channel spacing and channel count. *W_a_* represents the width of the input/output access waveguides of the AMMI devices. As *W_a_* is usually a few micrometers in width, tapered waveguides are utilized for adiabatic transmission and mode conversion between the access waveguides and single mode waveguides. The former is mainly limited by the minimum waveguide separation *x_min_* for avoiding the cross-coupling of adjacent waveguide channels, while the latter can be obtained from the available access waveguide window in the simulated field profile of Figure 10b. 

Following the design rules, the AMMIs are designed based on two considerations. Firstly, as the AMMIs are designed to be folded back to be alongside the MZI modulators, a footprint of 2–4 mm is acceptable for our transmitter design. Secondly, the insertion loss should be as small as possible while the required channel spacing and channel count are fulfilled simultaneously. As mentioned before, the 1-dB bandwidth of the apodized bidirectional grating coupler is about 40 nm. Thus, a channel spacing of 10 nm and channel count of 4 (halved to 5 nm and doubled to 8 by interleaved design) is preferred and chosen. To minimize the optical loss and crosstalk, the design parameters are fully explored by the Eigenmode Expansion (EME) [48] solver embedded in the commercially available software of Lumerical mode solutions. For fabrication convenience, all the waveguides of the AMMIs are designed with a ridge waveguide structure of 220 nm in height and 150 nm in etch depth, which is the same as that of the PN phase shifters. The optimized results for the design parameters of the AMMIs are listed in Table 4. Figure 10a shows the calculated field intensity profile of a single channel AMMI with an incident wavelength of 1550 nm. The high-fidelity self-imaging phenomenon can be clearly seen which indicates a low transmission loss. Figure 10c,d show the simulated optical transmission spectra of two 1 × 4 AMMIs. As can be seen, the insertion loss is about 0.52 dB, and the crosstalk between adjacent channels is below −23 dB.

The MZI optical interleaver is formed by an apodized bidirectional grating coupler connecting with taper waveguides, single mode waveguide arms and a 2 × 2 MMI coupler. The working principle is very similar to the MZI used for optical modulators. Ideally, if the waveguide loss and power combiner loss can be neglected and the fiber is in perfect coupling position, the optical transmission spectra of the MZI filter can be expressed as follows:

(17) $$\{\begin{array}{c} T_{1}\left(\lambda \right)=\frac{C\left(\lambda \right)}{2}\cdot \left[1+\cos\left(\varphi \left(\lambda \right)+\frac{\pi }{2}\right)\right]\\ T_{2}\left(\lambda \right)=\frac{C\left(\lambda \right)}{2}\cdot \left[1+\cos\left(\varphi \left(\lambda \right)-\frac{\pi }{2}\right)\right] \end{array}$$

(18) $$\varphi \left(\lambda \right)=\frac{2\pi N_{effw}}{\lambda }\cdot \Delta L$$

where the definition of the parameters in the equations are the same as mentioned before. In order to match the AMMIs and the MZI comb filter, the arm length difference (Δ*L*) of the MZI needs to be calculated accurately. By using the S-parameter matrices of the discrete optical components, the circuit-level simulation of the MZI is performed and the waveguide length difference is designed as 60.45 μm. 

**Figure 11:**
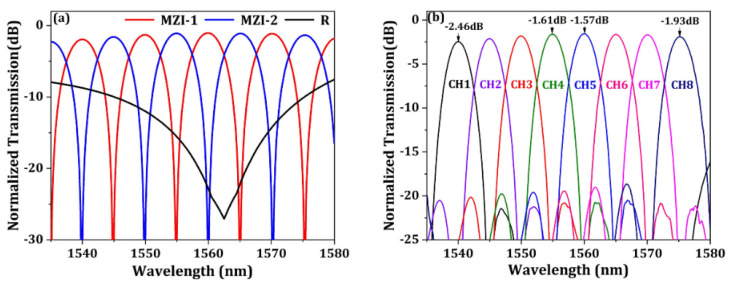
(**a**) The simulated optical transmission spectra of the MZI optical interleaver. (**b**) The simulated optical transmission curve of the eight output channels of the interleaved AMMI device.

Figure 11a shows the calculated optical transmission spectra of the MZI optical interleaver. MZI-1 and MZI-2 stand for the two complementary outputs of the MZI optical interleaver, while R represents the return loss of the device with the optical input from the grating interface. By connecting the MZI and the AMMIs in the circuit-level model, the optical spectra of the interleaved AMMI can be obtained as shown in Figure 11b. The insertion loss of the WDM device is (1.57–2.46) dB, which means the channel nonuniformity is below 1 dB. The average crosstalk between adjacent channels is about −23 dB. 

## 4. System Modeling and Performance

**Figure 12:**
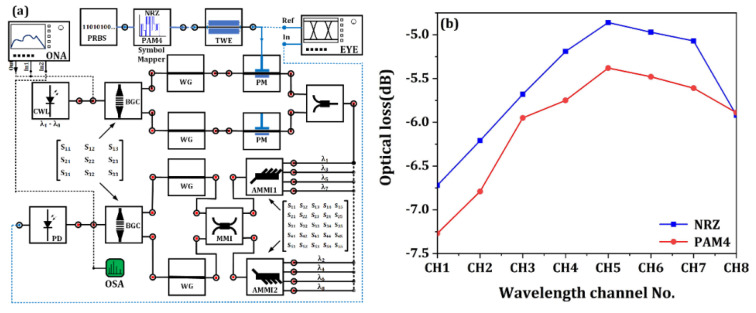
(**a**) Schematic diagram of the system-level simulation of the optical transmitter for eye diagram test and dynamic performance characterization. (**b**) The optical loss of the transmitter under NRZ modulation and PAM4 modulation format.

Based on the above modeling and simulation work of the discrete devices, the system-level simulation of the optical transmitter is carried out using the commercially available software of Lumerical Interconnect. The schematic diagram of the system modeling is shown in Figure 12a. As can be seen, the optical transmitter is characterized channel by channel using a tunable continuous wave laser (CWL). As mentioned before, two grating designs are used for the modulators. By substituting the S-parameter matrix of the bidirectional grating coupler (BGC), performance of different wavelength channels can be obtained. An optical network analyzer (ONA) is utilized to obtain the optical transmission spectra where the optical loss can be evaluated. For the dynamic performance, a PRBS generator, symbol mapper for NRZ and PAM4 modulation format and eye diagram analyzer (EYE) were added in the simulation for data transmission test. An optical spectrum analyzer (OSA) is also added to the optical output of the transmitter for frequency-domain analysis of the modulated optical signal. 

The optical loss of the optical transmitter determines the signal-noise ratio of the modulated signal and largely affects the complexity of the receiver circuit. Figure 12b shows the optical loss of the optical transmitter under NRZ and PAM4 modulation format. For the NRZ modulation format, the optical loss of the transmitter is (4.86–6.72) dB. For the PAM4 modulation format, the optical loss is (5.38–7.27) dB. Due to the bias condition difference, the optical loss of PAM4 modulation is about 1dB below that of NRZ modulation. Nevertheless, the optical loss is kept well below 8 dB for all wavelength channels, which is acceptable for the silicon transceiver applications. 

**Figure 13:**
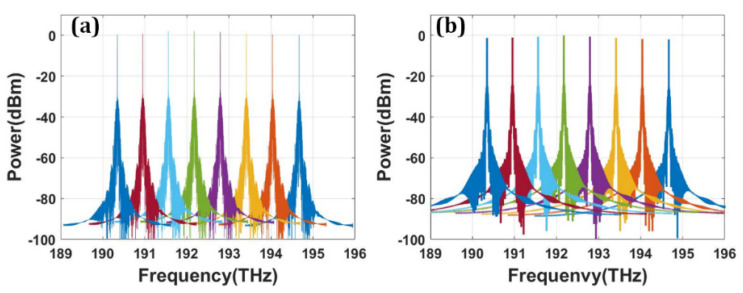
The calculated frequency spectra of the eight-channel modulated optical signals (**a**) with NRZ modulation format. (**b**) with PAM4 modulation format.

**Figure 14:**
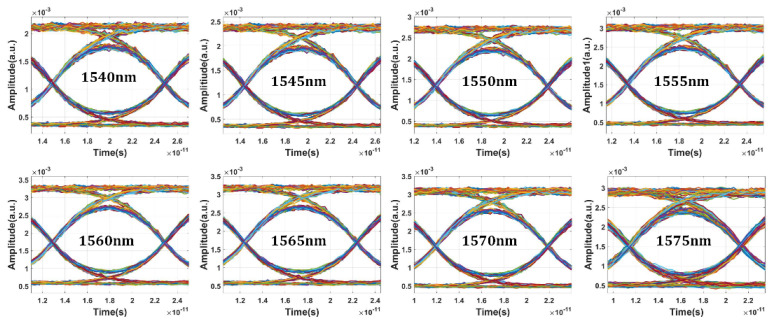
Simulated eye diagram results of eight channels of silicon photonic transmitter with NRZ modulation format of 100 Gb/s.

**Figure 15:**
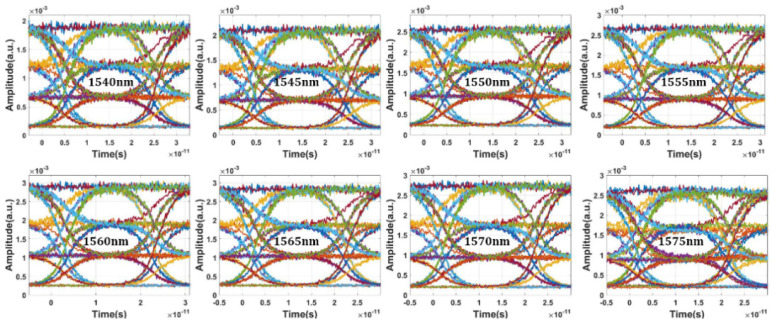
Simulated eye diagram results of eight channels of silicon photonic transmitter with PAM4 modulation format of 50 GBaud/s.

Figure 13a,b shows the calculated frequency spectra of the eight-channel modulated optical signals with NRZ and PAM4 modulation format. The modulated optical signal shows good uniformity between different channels. The channel isolation is below −60 dB for both modulation formats. Obviously, the signal integrity for 100 Gb/s NRZ signal is expected to be better than 50 GBaud/s PAM4 after optical filtering or demultiplexing. Figure 14 shows the NRZ modulation format eye diagram test results of the optical transmitter with data rate of 100 Gbps and input optical power of 10 dBm. Benefiting from the high performance of the bidirectional grating couplers, clear open eyes were obtained for eight channels. The modulation depth is about 6.7 dB. By replacing the NRZ signal generator with a PAM4 signal generator, the eye diagram test of PAM4 modulation format can be carried out. Compared with the NRZ signal, the optical signal with PAM4 pattern is more sensitive to noise. Thus, the requirement for signal quality is more rigorous. To achieve good linearity and symmetry of eye diagrams, bias conditions for working wavelength and driving voltage levels are carefully chosen. Figure 15 shows the simulated eye diagram test results of eight channels. Small ripples can be clearly seen, which is mainly due to the optical and electrical reflections within the photonic link. Notably, the eye diagrams do not show obvious deterioration at the edge channels. The extinction ratio is about 4.54 dB. For PAM4 eyes, the level separation mismatch ratio (RLM) is an important concern which is directly related to the BER. The RLM of eight-channel PAM4 eye diagrams are analyzed. According to our calculations, the RLM is above 0.94 for all the wavelength channels, which should be able to meet most of the PAM4 specifications.

**Figure 16:**
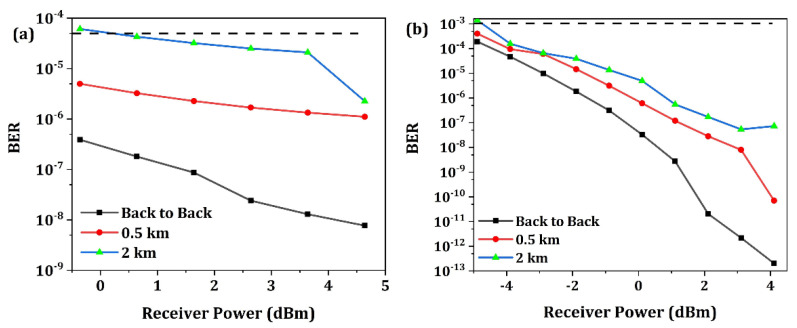
(**a**) Calculated curve of BER versus the received optical power for 100 Gb/s OOK signal transmission. (**b**) Calculated curve of BER versus the received optical power for 50 Gbaud/s PAM4 signal transmission.

The BER test simulations were carried out for both NRZ and PAM4 modulation format signal transmission. Figure 16a,b shows the measured curve of BER versus the received optical power (ROP) for 100 Gb/s NRZ and 50 Gbaud/s PAM4 signal transmission, respectively. The ROP is equal to the input laser power minus the total optical loss of the optical transmitter chip. Performance under different transmission lengths is investigated, including back-to-back (0 km), 0.5 km and 2 km single mode fiber. Here, a model of single mode fiber G.652 is utilized with dispersion characteristics. For OOK and PAM4 signal, FEC [49,50] threshold of 5 × 10^−5^ and 1 × 10^−3^ are assumed for error-free transmission, respectively. Thus, it can be seen that the minimum achievable ROP for 500 m transmission is about −1 dBm and −5 dBm for 100 Gb/s OOK and 50 Gbaud/s PAM4, respectively. 

The power consumption of the optical transmitter is of great concern in real applications. In our estimation, the total power consumption is divided into three parts. Firstly, the modulators are power hungry devices. According to our simulations, the PN junction capacitance of 2 mm phase shifter length is about 0.44 pF. Since the modulators are working in push-pull differential mode, the capacitance of a single modulator would be doubled as 0.88 pF. According to the equation of modulator power consumption:

(19) $$E=C\cdot V_{pp}^{2}/4$$

Here *V_pp_* of 2 V is utilized in this design. Then the power consumption of a single modulator is estimated to be 0.88 pJ/bit. The power consumption of 800 G optical modulation is about 0.704 W. Secondly, the thermal tuning functions are added for setting the operation point of the modulators, which contributes to the total power consumption. Assuming that the length of thermal tuning functions is about 100 μm, the resistance of TiN thermal heater is about 210 Ω. At 3 V, the power consumption of single channel thermal tuning is about 43 mW, and the total power consumption of thermal tuning is about 0.35 W (including the possible thermal tuning in the WDM function). Finally, although lasers are not part of silicon photonics chips at present, they contribute to the total power consumption of silicon photonic transmitter as well. According to the datasheet of a DFB laser product of Lumentum corporation, the typical power consumption of a single laser is about 90 mW, which corresponds to an output optical power of 11.5 mW. This is fairly enough for our transmitter power budget. Therefore, the total power consumption of eight lasers is 0.72 W at maximum. The power consumption of the 800 Gbps WDM silicon photonic transmitter is about 1.77 W in total. Other power consumptions for ASICs and TEC are beyond our discussions here. Considering the power consumption standard of 800G silicon photonic transceiver modules is 16 W, the total power consumption of our transmitter chip is acceptable in general.

## 5. Conclusions

In conclusion, an 800 Gbps silicon photonic transmitter with surface-normal optical interfaces is designed and numerically demonstrated, which is comprised of eight Mach–Zehnder optical modulators and an interleaved AMMI WDM device. The interleaved AMMI is built up by a Mach–Zehnder optical interleaver and two 1 × 4 AMMIs. With apodized bidirectional grating coupling technology, perfectly vertical optical coupling with about −0.5 dB coupling loss can be achieved by simulation. Mach–Zehnder E-O modulators and optical interleaver can be built up by using the 3-dB power splitting behavior of the bidirectional grating couplers. To achieve a high performance optical transmitter, the optical devices are fully optimized with simulations. By building up the circuit-level model of the optical transmitter, both the static and dynamic performance characterization were carried out. The overall optical loss of the optical transmitter is (4.86–6.72) dB and (5.38–7.27) dB for eight wavelength channels with NRZ and PAM4 modulation format, respectively. Eye diagram test results show that all the wavelength channels can support a 100 Gb/s NRZ data rate or 50 GBaud/s PAM4 symbol rate and the BER results for two modulation formats are acceptable for optical communication standards.

## Figures and Tables

**Table 1:** The specific parameters of grating period and duty cycle.

H = 220 nm, h = 160 nm, d = 230 nm											
ABG1	P_i_(nm)	579	560	568	575	600	595 *	589 *	592 *	595 *	598 *
	FF_i_	0.38	0.38	0.38	0.35	0.23	0.47 *	0.51 *	0.44 *	0.37 *	0.23 *
ABG2	P_i_(nm)	579	560	568	627	600	595 *	589 *	592 *	595 *	598 *
	FF_i_	0.38	0.38	0.38	0.30	0.23	0.47 *	0.51 *	0.44 *	0.37 *	0.23 *

* The parameters of subwavelength grating structure: FF_s_ = 0.67, P_s_ = 400 nm.

**Table 2:** The performance comparison between our design and other fiber-chip grating couplers.

Reference	CA *	CE * (dB)		BW * (nm)		CD * (nm)	FS *	
		Meas	Simu	−1 dB	−3 dB		E *	D *
[37]	15°	−1.2	−1.1	78	-	45	3	3
[38]	-	−1.3	−1.1	-	52	110	2	1
[39]	0.5°–2.8°	-	−1	>90	-	<100	1	1
[40]	22°	−2.7	−2.2	-	62	193	2	1
[41]	17°	-	−0.25	-	-	100	2	1
[42]	0°	−1.5	-	-	49	81	2	1
[43]	19°	−0.36	−0.22	-	100	<100	3	3
This work	0°	-	−0.54	37	75	130	1	2

* CA: coupling angle, CE: coupling efficiency, BW: bandwidth, CD: critical dimension, FS: fabrication steps, E: etching, D: deposition.

**Table 3:** The design parameters of the PN phase shifter.

W_rP_ = 0.3 μm, W_rN_ = 0.1 μm, W_s_ = 0.125 μm						
Doping region	P++	N++	P+	N+	P	N
Concentration cm^−3^)	1 × 10^20^	1 × 10^20^	4 × 10^18^	4 × 10^18^	3.5 × 10^17^	6 × 10^17^
Span (μm)	4.75	4.75	5.625	5.625	0.425	0.425

**Table 4:** Optimized design parameters of the two 1 × 4 AMMIs.

*W_a_* = 8 μm, *W_m_* = 23 μm, *θ* = 17.23°, *X_min_* = 1 μm					
AMMI1	*λ_i_* (nm)	1540	1550	1560	1570
	*L_i_* (μm)	3484	3454	3424	3394
AMMI2	*λ_i_* (nm)	1545	1555	1565	1575
	*L_i_* (μm)	3469	3438	3409	3380
